# Balloon-Atrioseptostomy in Small Children Using an Embolectomy Catheter: Preliminary Data

**DOI:** 10.1155/2023/9920336

**Published:** 2023-07-26

**Authors:** R. Dalla Pozza, M. Hermann, N. A. Haas

**Affiliations:** Division of Pediatric Cardiology, Ludwig Maximilian-University of Munich, Munich, Germany

## Abstract

Interventional treatment of restrictive atrial septal defects in complex heart disease is considered state-of-the-art therapy up to date. Nevertheless, dedicated balloons are lacking so far, as several products have been withdrawn from the market. We report on off-label use of a balloon embolectomy catheter used successfully in a preterm patient and discuss whether this device might be used in other patients as well as it seems to be promising due to its shape and versatility.

## 1. Introduction

Interventional balloon-atrioseptostomy (BAS) is considered the treatment of choice in neonates with restricted atrial shunting and complex congenital heart defects like transposition of the great arteries (TGA) or Hypoplastic left heart syndrome [1–5]. It is also important for new interventional strategies designed for the treatment of complex congenital heart defects in preterm babies. Despite those new developments, several products for balloon-atrioseptostomy have been withdrawn from the market, so right now the only available balloon is the atrioseptostomy catheter “Z-6” (NuMED Inc, Hopkinton, NY, USA). However, based on its design bedside echocardiography-guided BAS may be difficult in small patients and many paediatric cardiologists prefer performing such intervention in their catheterization laboratory using radiation: that is, the angle of the catheter tip of 35° may be too small for the atrial anatomy in small neonates with a body weight less than 2 kg to pass the atrial septum easily. In addition, the use of a 0.021″ guidewire recommended for introducing the balloon and passing the atrial septum may be more difficult in echocardiography-guided intervention. Finally, the use of a 6 Fr introducer recommended by the manufacturer may facilitate vascular complications in small neonates.

**Figure 1:**
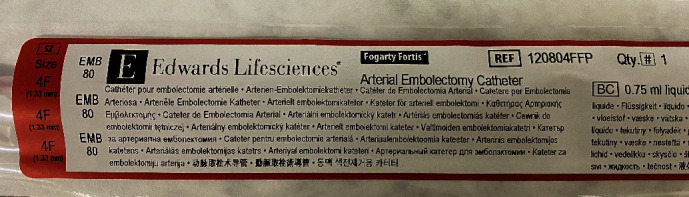
Edwards Lifesciences Fogarty Fortis Catheter Specs.

We report on a balloon embolectomy catheter (Edwards Fogarty Fortis Arterial Embolectomy Catheter, REF. # 120804FFP, Edwards Lifesciences Inc., Irvine, CA, USA, Figure 1) used off-label successfully in a small preterm baby, which seems to show promising properties for the use in small patients.

## 2. Case Description

**Figure 2:**
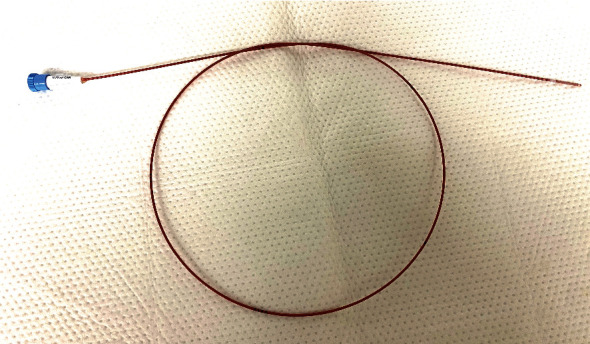
Regular shape of the catheter with a straight tip, 80 cm long.

**Figure 3:**
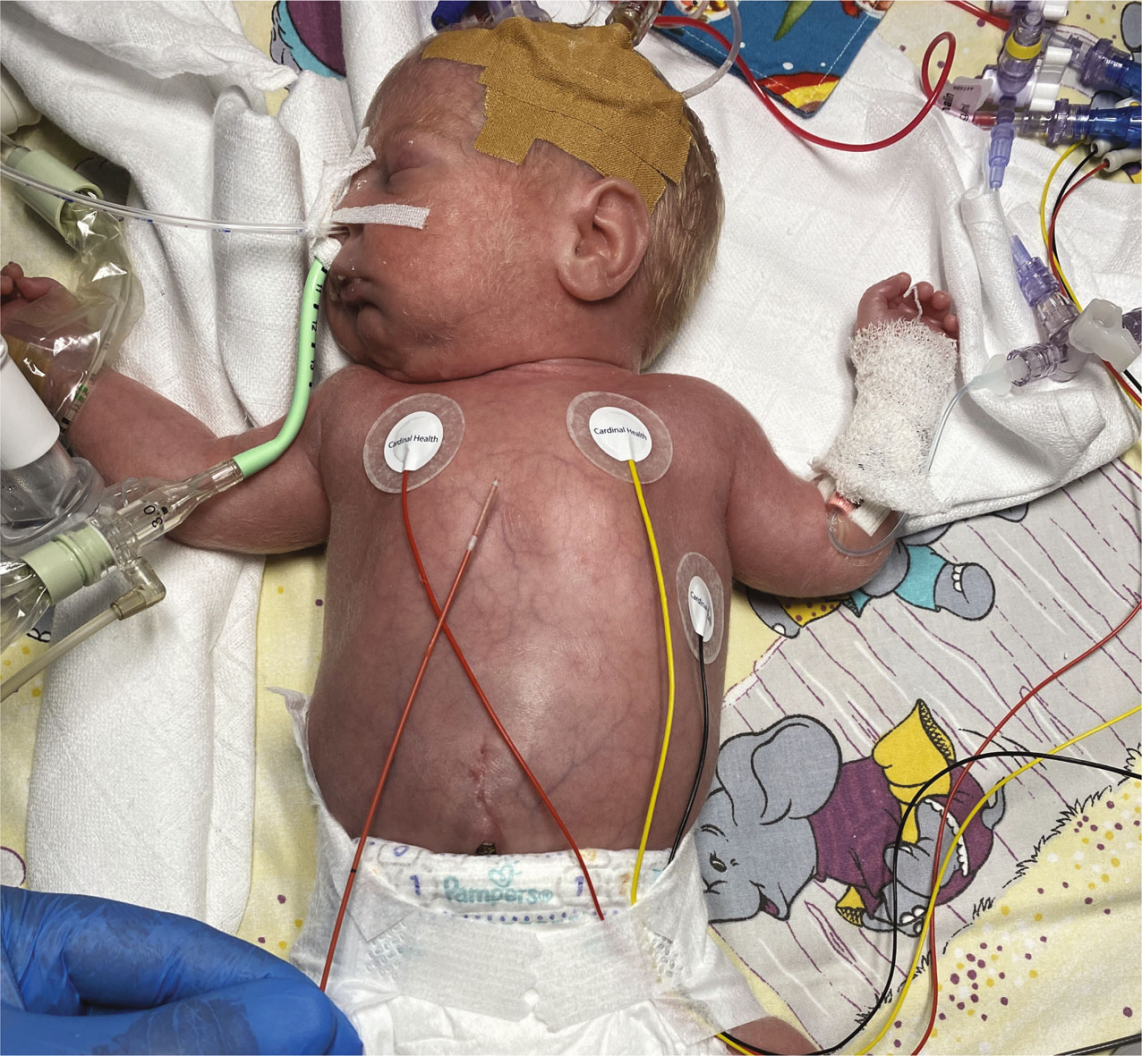
Patient (32 weeks of gestation, 1.2 kg) with TGA, VSD, and restrictive ASD. Edwards Fogarty Fortis Catheter with straight tip.

**Figure 4:**
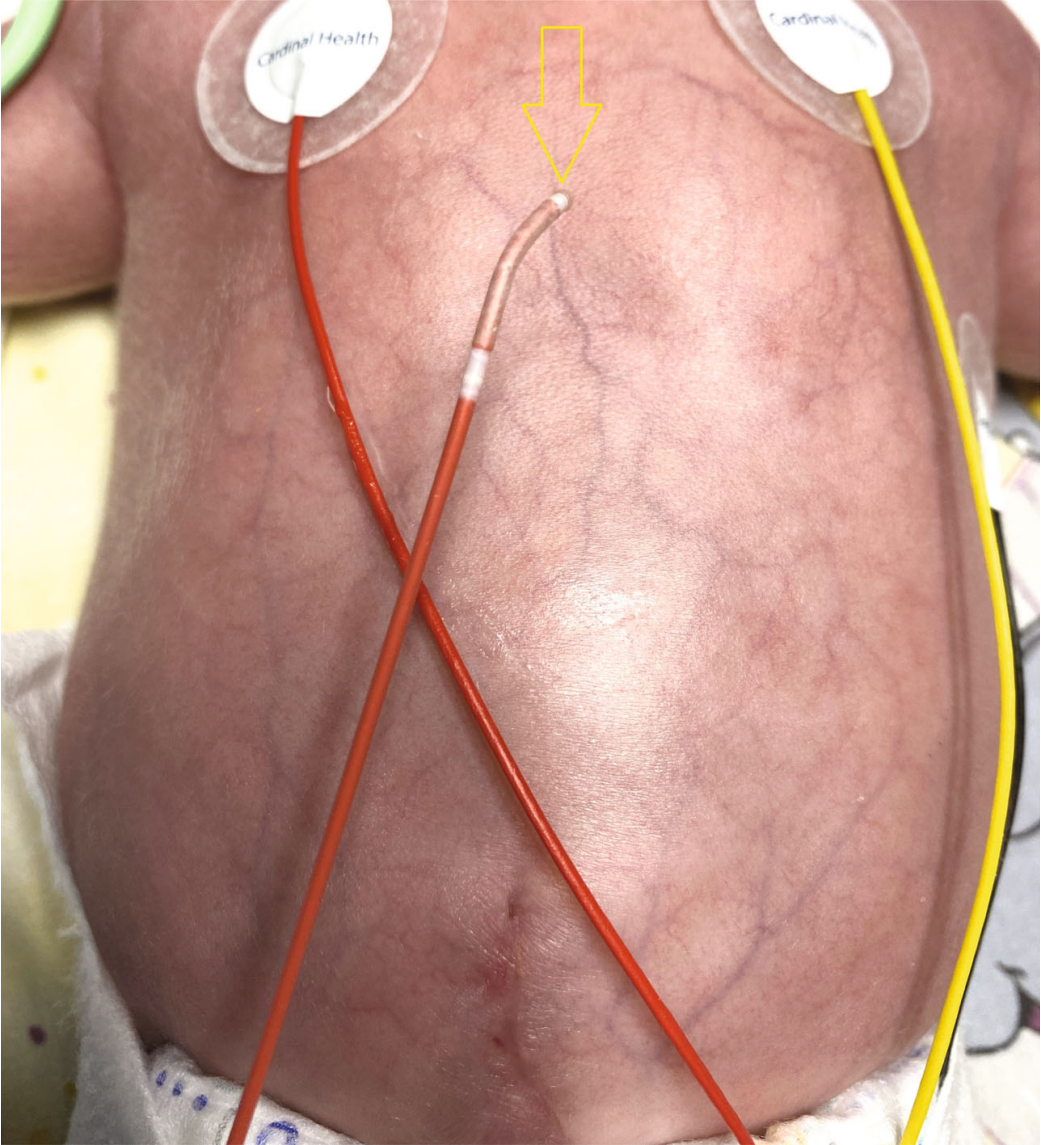
Individually modified catheter tip according to the atrial anatomy of the patient (arrow).

**Figure 5:**
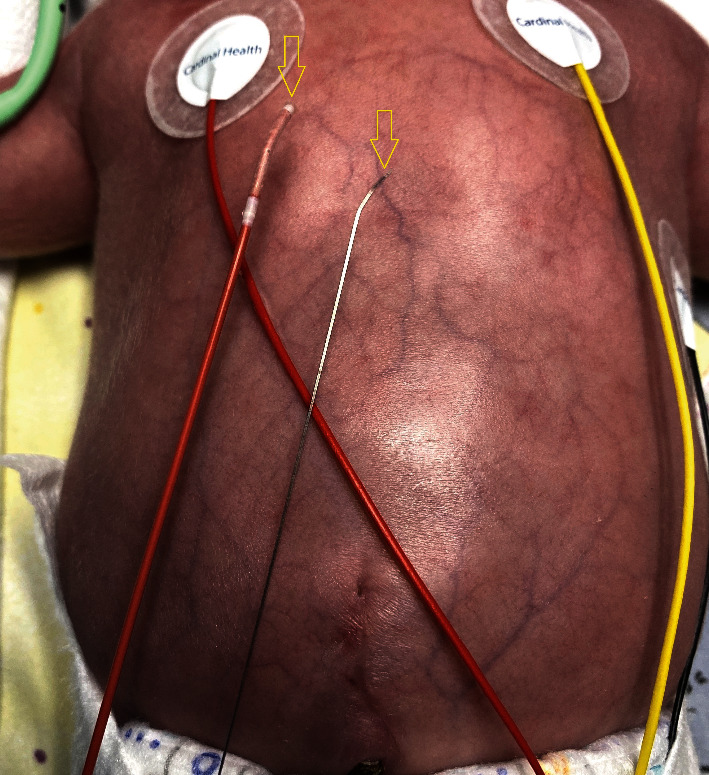
Catheter and stylet shaped (arrows).

**Figure 6:**
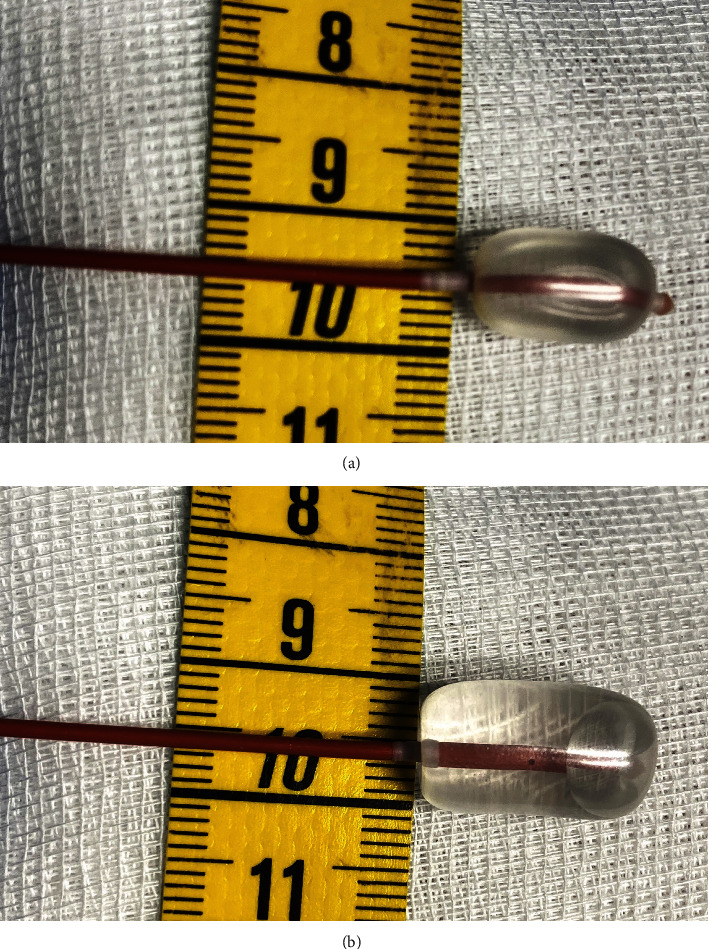
(a) Balloon with a volume of 0.75 cc saline inflated and a diameter of 0.6 cm. (b) Balloon with a volume of 1.5 cc of saline inflated and a diameter of 1.0 cm.

**Figure 7:**
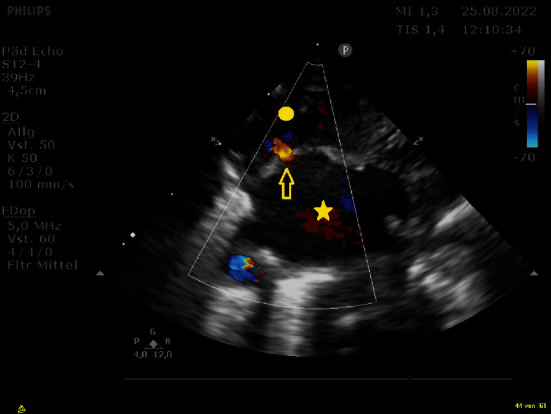
Echocardiography of atrial anatomy of the patient showing a severely dilated left atrium (star), a restrictive atrial septal defect (arrow), and a small right atrium (dot).

**Figure 8:**
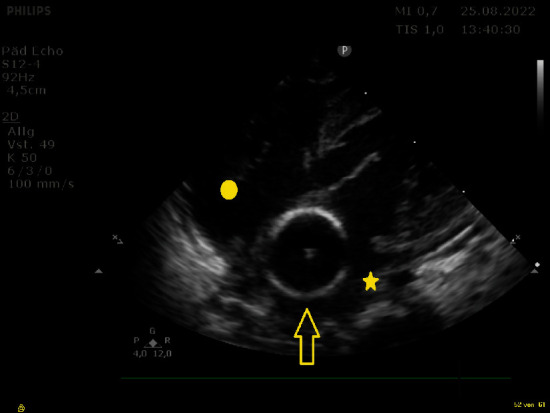
Echocardiography of the same patient with the balloon inflated (arrow) in the left atrium (star). Dot: right atrium.

**Figure 9:**
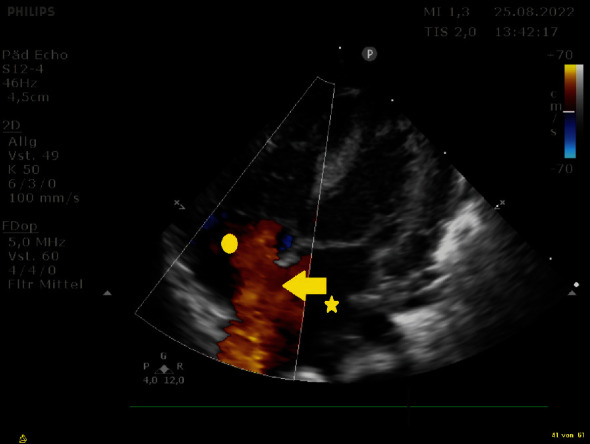
Echocardiography after BAS: a large ASD with left-to-right-shunting is present (arrow); the left atrium is decompressed (star) and the right atrium is larger (dot).

In our patient (4 weeks, 1.2 kg, and 32 weeks of gestation), TGA with ventricular septal defect (VSD) and atrial septal defect (ASD) was detected by echocardiography. After the first four weeks of life, the ASD became restrictive (diameter: 2 mm), and progressive left heart volume overload caused left heart failure associated with inadequate systemic perfusion, edema formation, and cyanosis. After intubation, the patient was transferred to our institution. Mechanical ventilation failed to resolve cyanosis (O_2_-saturation 65%, FiO_2_ 1.0), and bedside echo-guided BAS was performed. We used an Edwards Fogarty Fortis Balloon Embolectomy Catheter and a Terumo 5 Fr radial sheath (the manufacturer recommends a 4 Fr sheath, but in our experience the balloon, when deflated after intervention, needs a 5 Fr sheath for extraction) with access from the right femoral vein (Figure 2). The catheter is of a single-lumen design with a stylet inside, but without an end-hole (Figures 3 and 4). The stylet allows for manual modification of the tip shape of the catheter (Figure 5). By that, it could be adapted to the anatomic situation of our patient. After that, the catheter could be easily advanced with the stylet inside from the right femoral vein to the left atrium without the need for guidewires. After the removal of the stylet and balloon inflation with 0.75 cc of saline (Figure 6), BAS was performed without any complication. On echocardiography, the ASD seemed to be too small again (Figure 7), and a second and third BAS were performed by inflating 1 and 1.5 cc of saline into the balloon, respectively (Figures 6 and 8). After that, a 5 mm wide ASD was seen on echocardiography, and the intervention was stopped (Figure 9). The time of intervention was ten minutes. Immediately thereafter, the O_2_-saturation improved to 85% and echocardiography showed an ASD of 5 mm with left-to-right shunting. Systemic perfusion, as well as O_2_-Saturation, remained stable so the patient lost about 300 g of edema during two days.

## 3. Discussion

We think that the catheter used in this situation is especially useful for small patients and for patients with complex cardiac defects. Even if designed for arterial embolectomy, it can be adapted to special anatomic situations by simple tilting of the catheter tip. Thus, bedside intervention may be facilitated avoiding transport to the catheterization laboratory and radiation. In addition, the balloon is very extensible: we tested the diameter of the balloon by inflating increasing amounts of fluid: from 0.75 cc and 0.6 cm diameter, we were able to inflate up to 1.5 cc for a diameter of 1.0 cm (however, the instructions for use of the manufacturer allow only 0.75 ml of volume to be inflated). Thus, several BAS may be feasible with increasing diameters of the balloon in the individual patient according to special anatomic and pathophysiologic situations. It should be noted that there may be special situations like unusual vascular access from the jugular vein in whom this catheter does not facilitate BAS. In addition, caution should be used to avoid balloon rupture, which may render catheter extraction impossible and expose the patient to possible embolism. Thus, dedicated informed consent for off-label use has to be obtained. Nevertheless, if those pitfalls have been addressed, we believe that the device presented may be very useful in experienced hands.

In conclusion, based on the need for alternatives to balloons for balloon-atrioseptostomy withdrawn from the market, we successfully used an Edwards Fogarty Fortis Arterial Embolectomy balloon catheter for bedside, echo-guided intervention in a 1.2 kg preterm baby with TGA, VSD, and restrictive ASD. We would like to present this catheter for such interventions, especially in small preterm babies in whom difficult atrial anatomy or hemodynamic instability may be particularly challenging.

## Data Availability

Data supporting this research article are available from the corresponding author or first author on reasonable request.
